# Can home care work be organized to promote musculoskeletal health for workers? Study protocol for the Norwegian GoldiCare cluster randomized controlled trial

**DOI:** 10.1186/s12913-022-08916-0

**Published:** 2022-12-07

**Authors:** Fredrik Klæboe Lohne, Marius Steiro Fimland, Andreas Holtermann, Svend Erik Mathiassen, Heike Fischer, Trine Minde Gellein, Skender Redzovic

**Affiliations:** 1 Department of Neuromedicine and Movement Science, Faculty of Medicine and Health Sciences, NTNU Norwegian University of Science and Technology, Trondheim, Norway; 2 Unicare Helsefort Rehabilitation Centre, Rissa, Norway; 3 National Research Centre for the Working Environment, Lerso Parkalle 105, DK-2100 Copenhagen, Denmark; 4 Centre for Musculoskeletal Research, Department of Occupational Health Science and Psychology, University of Gävle, 80176 Gävle, Sweden; 5 Trondheim municipality, Bergheim home care service, Postboks 2300 Torgarden, 7004 Trondheim, Norway

**Keywords:** Goldilocks work principle, Workplace health promotion, Occupational health, Musculoskeletal pain, Lower back pain, Neck pain, Shoulder pain, Cluster-randomized controlled trial

## Abstract

**Background:**

Home care workers perform physically strenuous tasks, in particular when handling patients with high care demands. Thus, musculoskeletal pain and sick leave is greater in this group than in the general population. To ease these issues, we will implement a Goldilocks Work intervention (GoldiCare), redistributing schedules between workers to achieve a “just right” weekly structure of physical work that can promote health. This protocol paper describes the content, design, implementation and evaluation of the cluster randomized controlled trial of the GoldiCare intervention in home care.

**Methods:**

The cluster randomized controlled trial is a 16-week workplace organizational intervention implemented through operations managers at the home care units. The operations managers will be introduced to the Goldilocks Work Principle and a GoldiCare tool, to assist the operations managers when composing a “just right” distribution of work schedules throughout the week. The GoldiCare tool provides an overview of the physical strain for each shift, based on the number of patients and their need for care. We expect to include 11 units, which will be randomized to either intervention or control at a 1:1 ratio. Home care workers assigned to the control group will continue to work as normal during the intervention period. Musculoskeletal pain in neck/shoulder and lower back will be the primary outcomes and we will also evaluate the composition of physical behaviors as well as fatigue after work as secondary outcomes. We will collect data using (1) daily questions regarding musculoskeletal pain and fatigue after work, (2) 7 days of objective measurements of physical behavior, (3) questionnaires about the participant’s characteristics, health, and workplace psychosocial stressors and (4) information on the implementation of the GoldiCare tool. In addition, a process evaluation will be conducted using focus group discussions and individual interviews.

**Discussion:**

Due to the increasing aging population in need of care, measures that can improve the health of home care workers are paramount for the sustainability of this sector. This organizational intervention is based on information available nation-wide, and therefore has the potential to be scaled to all municipalities in Norway if proven effective.

**Trial registration:**

This clinical trial was registered on 08/05/2022 under NCT05487027.

## Background

Home care workers care for patients living at home. The workers need to adapt to the patients’ health, functional abilities and living circumstances, such as crowded and small residences that are frequently insufficiently equipped for the required care tasks [1]. As a result, home care workers may spend considerable periods of time in awkward postures, often involving pulling and pushing while caring for patients [1, 2], and home care workers often find their work physically strenuous [3–5]. As a likely consequence, home care workers often report musculoskeletal pain and sick leave; as illustrated by their 11% sick leave rate, almost double the national average [6–8]. As the number of elderly aged above 65 is forecasted to double by 2050, and that of those aged above 80 years to triple [1, 9, 10], the home care workers are expected to play a crucial role in future health care service, as the home care sector offers a cost-effective solution for providing care [10–12]. However, to keep up with the increasing demands, the employees working in this sector must be healthy and working.

A way of improving employee health and limit sick leave might be to redesign work according to the Goldilocks Work Principle, which aims to make the physical work demands “just right.” [13, 14] A just right task distribution entails that workers are exposed to neither too little nor too much physical strain; both scenarios may lead to unfavorable exposures and thus suppress health [15]. Moreover, the vision of the Goldilocks Work Principle is that the just right distribution not only prevents poor health, but also promotes workers’ health and capacity. While the feasibility of Goldilocks interventions have been investigated in other sectors, such as child care [16] and manufacturing [17] no randomized controlled trial has been conducted in home care to date.

As a part of an earlier investigation, we noted a high prevalence of musculoskeletal pain in the neck/shoulder area (36%) and lower back (34%) among home care workers in Trondheim, Norway [2]. Further, we found long durations of awkward postures such as forward trunk inclination and arm elevation during the working day [2] which is documented to increase the risk of sick leave [18, 19]. In addition, the strenuous tasks were unevenly distributed among workers and a substantial proportion of employees were exposed to very high levels of physical strain [2], likely to lead to a substantial risk of musculoskeletal pain and sickness absence. Available evidence indicates that strenuous activities such as pulling, pushing, lifting, and leaning forward are frequently performed by home care workers caring for patients with extensive care needs [20, 21]. Patients who are admitted into the Norwegian home care system, are assessed by a trained nurse in terms of their care needs based on the International Classification of Functioning, Disability and Health system developed by WHO [22, 23], resulting in an activities of daily living (ADL) score. The total score is the average of five subcategories; 1) social function, 2) cognitive function, 3) ability to take care of their own health, 4) domestic responsibilities, and 5) self- care. Home care workers are mainly responsible for assisting with self-care and therefore we investigated if patients’ self-care ADL (hereafter referred to as “ADL”) score was associated with the physical work demands of home care workers. We found patients with a high ADL score (4–5, out of 5) to require the home care workers to stand for relatively longer durations, compared to when caring for low ADL patients (1–2, out of 5). Previous studies and our pilot investigations imply that ADL scores may potentially act as a proxy for the physical work demands put on the home care workers. Due to the handling of patients being a major source of exposure to physical work demands, the ADL scores may be used to redesign the uneven distribution of physical work demands into being “just right”, which could improve the musculoskeletal health of home care workers.

In this protocol paper, we describe a cluster randomized controlled trial developed to evaluate the effectiveness of a Goldilocks Work intervention in promoting musculoskeletal health among home care workers.

### Trial design

This Goldilocks work intervention will be implemented as a cluster randomized controlled trial with two parallel groups—a no-intervention control arm and an intervention arm, with clusters allocated to the control and the intervention groups in a 1:1 ratio.

## Methods—participants, interventions, and outcomes

### Study setting

The intervention will be conducted in Trondheim municipality, and an invitation for voluntary participation was distributed to all 13 home care units operating in this area. Eleven positive responses were received. Each home care unit in Trondheim municipality employs 23–95 home care workers providing health services to patients living in their private homes within a defined geographical area. Each unit is organized in teams, which are responsible for a smaller section of the unit, often demarcated by geography. This organizational structure ensures home care workers’ familiarity with the patients, thereby increasing effectiveness, efficiency, and work quality. Seeing familiar home care workers every day also gives structure and reassurance to the patients. The operations manager distributes the daily working lists defining the assignments of each home care worker and an approximate timeframe for the visit to each patient.

### Eligibility criteria

As the intervention is conducted at the organizational level, all employees working at a unit randomized to the intervention arm will be affected regardless of their participation in study-specific measurements. However, all home care workers with ≥50% employment will be invited to participate in baseline measurements unless they are pregnant, have a fever, are allergic to the tape used to attach the sensors, or have a physical impairment that precludes normal physical activity. Home care workers volunteering to participate will be required to read and sign an informed consent form before enrolment in the measurements, clearly explaining their rights in accordance with the Declaration of Helsinki.

### Intervention

#### Development

The intervention was developed in accordance with the intervention mapping strategy [24]. The first step involved formulating a “logic model of the problem” describing the population at risk and the context. Next, the “logic model of change” was developed, whereby the intervention outcomes and objectives were defined based on the findings obtained in the first step. In the last step, an intervention comprising both evidence- and theory-based methods was designed, securing that it will be appropriate for the given context and problem.

#### Logic model of the problem

To measure the physical behavior (e.g., sitting, standing, walking and running etc.) and its intensity during a typical workday, an observational study involving 6 units in Trondheim municipality was conducted using accelerometers and heart rate monitors [2]. Analysis of the data revealed a considerable variation between home care workers in the time spent in awkward postures, i.e. arm elevation and forward trunk inclination. Also, a high proportion of workers reported long-term (≥3 consecutive months in the past year) neck/shoulder pain (36%) and lower back pain (34%) [2].

#### Logic model of change, program outcomes and objectives

A participatory approach was adopted to identify potential areas of focus for the intervention, with eight home care workers and five operations managers taking part in digital workshops. They communicated that the patient visits were unevenly distributed among the home care workers, in terms of patients requiring extensive care and light care. We examined the patients’ ADL scores, assuming that they reflect the level of care for the patient, and thus can serve as a proxy for the physical work demands of the home care worker [20, 21]. To check this assumption, we used objective physical activity data from a feasibility study in home care and associated them with ADL scores of the patients visited during selected shifts. The results confirmed that workers caring for patients with a higher ADL score had, on average, a higher physical work load compared to when looking after patients with lower ADL scores (unpublished data).

**Fig. 1:**
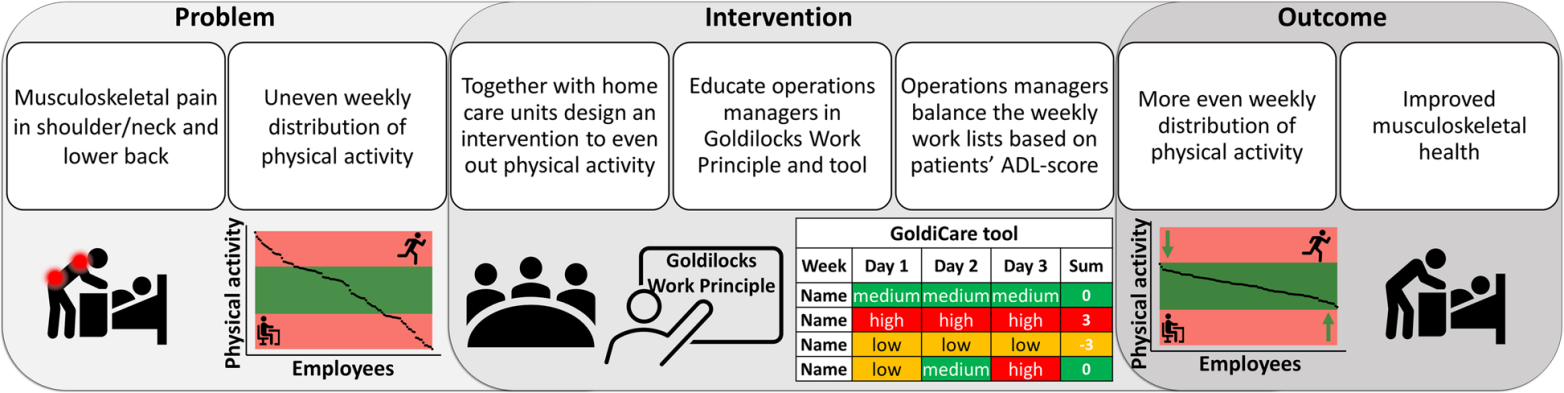
Program logic explaining the problem at hand, the intervention aims, and the expected outcome of the intervention. *ADL* Activities of Daily Living

Based on these findings, our intervention objective was to promote musculoskeletal health in the neck/shoulder area and in the lower back by ensuring that home care workers do not receive disproportionate numbers of patients with a high ADL score within a week, as shown in the program logic model in Fig. 1. A variation for the individual home care worker between working lists with many high ADL patients and working lists with few or no high ADL patients, offers an alternation between higher and lower strain. The choice to aim for a weekly just right distribution of physical strain instead of a daily just right distribution was a result of the working lists being designed and scheduled based on several factors that could not be controlled as a part of the intervention, such as geography, technical skills, qualifications, and time management. Thus, changing the working lists within a workday was not a feasible strategy.

#### Program design and production

The “GoldiCare” intervention described here was developed in accordance with the Goldilocks Work Principle, which aims at improving worker health by re-designing work at the organizational level without compromising productivity [13]. The intervention was also developed in cooperation with the municipality as a nurse from one of the participating home care units is a member of the research team, contributing with important knowledge and experience of conditions in home care.

As part of the intervention, each operations manager will be provided with a “GoldiCare tool” developed in Microsoft Excel to support a more even distribution of high ADL patients among home care workers. The GoldiCare tool was inspired by a similar Goldilocks Work intervention implemented in Denmark [17]. The GoldiCare tool was tested in a feasibility study (unpublished), and an adjusted version was adopted for the present intervention. When using the GoldiCare tool, the operations managers will work towards achieving an even distribution between low and high strain days as days with medium strain are regarded to be “just right”. Operations managers define the strain level for the working day, based on the number of patients with high ADL scores on the working list. Since the total number of high ADL patients differs between units, the average physical work demand, as expressed by the ADL scores, differed between units. The GoldiCare tool eventually results in a weekly summary for each employee based on the composition of high, medium and low strain days throughout the week, and serves to inform the operations managers of any major differences between home care workers in the allocation of high ADL patients. The weekly summary per employee is used to obtain an overall summary for the entire unit, reflecting how well the operations managers implemented the intervention at the unit. Thus, the GoldiCare tool serves several objectives: [1] operations managers can use it to review previous and planned shifts, and can thus better distribute physically strenuous work in line with the Goldilocks Work Principle to give a sustainable practice in the long term, [2] both researchers and operations managers can gain insight into the level of physical strain among home care workers in a working week, and [3] both researchers and operations managers can quantitatively assess home care unit’s compliance with the intervention.

#### Implementation plan

The intervention described above will be implemented in a two-step process. In the first step, lasting for a total of 3 weeks, operations managers will get a thorough and personalized introduction to the GoldiCare intervention, including its aims and underlying scientific basis, and the usage of the GoldiCare tool. During this introduction, the percentage of high ADL patients required to define a working list as ‘high strain’ will be determined by the operations manager and the researchers together. In the two subsequent weeks, the operations managers will use the GoldiCare tool in their planning, and the researchers will visit each unit to assist and give guidance if needed. The second step will run throughout the intervention period, allowing the operations managers to use the GoldiCare tool in practice and receive timely feedback from the researchers. The feedback will include how the unit’s performance of the week compared to previous weeks, and the summaries of individual home care workers. The operations managers will be encouraged to contact the researchers at any point during the intervention period if problems or questions arise. To allow researchers an overview of the usage, the GoldiCare tools will be saved and edited in a cloud-based server.

#### Outcomes

##### Primary outcomes

- Difference between intervention and control group in the change from pre- to post-intervention of lower back pain intensity.
- Difference between intervention and control group in the change from pre- to post-intervention of shoulder/neck pain intensity.

Both primary outcomes will be measured after each working day during 1 week at baseline and 1 week at the end of the intervention, using a 11-point numeric scale (0–10).

##### Secondary outcomes

- Difference between intervention and control group in the change from pre- to post-intervention of the composition of awkward postures (arm elevation and trunk inclination), assessed by accelerometers.
- Difference between intervention and control group in the change from pre- to post-intervention of the composition of physical behavior (standing, sitting and physical activity), assessed by accelerometers.
- Difference between intervention and control group in the change from pre- to post-intervention of fatigue, measured after each working day for 1 week using a 11-point numeric scale (0–10).
- Compliance with the intervention, as determined by the units’ weekly summary. For this purpose, the weekly summary of variables calculated in the GoldiCare tool will be compared to the baseline.

### Sample size

Preliminary mapping of the Trondheim home care sector indicated that 440 home care workers meet the ≥50% employment criteria. As our previous investigation of the home care sector [2] had a 58% participation rate, we expect that 255 home care workers would be recruited for baseline measures if the invitation is distributed to all 440. Assuming a 20% attrition rate, 204 home care workers will complete both baseline and post-intervention measurements, which would result in 102 participants in both the control and intervention arms.

We do not expect different effects in the clusters of intervention and control units since the clusters are made based on feasibility and convenience. With 102 participants in each arm, and an alpha of 5%, we have an 80% chance of detecting an effect size (i.e. an intervention effect) corresponding to a Cohen’s d of 0.39. As no data on the variance in pain scores following a working day in the home care sector currently exist, we were guided by the variance (10.6) from a Goldilocks Work intervention in an industry setting [25], based on which a Cohen’s d of 0.39 would correspond to an intervention effect of 1.27 points. Given that a 1.74 difference on the numeric rating scale between control and intervention is recognized as clinically significant [26, 27] we find that a sample comprising 204 participants is sufficient to detect a clinically meaningful difference.

### Recruitment strategy

Involving managers is vital for a successful implementation of an organizational intervention in the home care sector [28]. Therefore, during the development phase of the project, representatives from the research group participated in three meetings with the unit leaders. To maximize the participation rate when assessing physical behavior and musculoskeletal pain, we visited each unit leader to present the intervention, aiming to generate a positive attitude toward the project, which would hopefully be transferred to the workers. During these visits, leaders also advised on the best implementation and recruitment strategies. In addition, to better inform the home care workers of the intervention and associated measurements, researchers will visit each unit and discuss these aspects with the home care workers, who will also receive flyers with short and concise information about the measurement procedures and the equipment involved. At the time of submission, participant recruitment is ongoing.

## Methods – assignment of interventions

### Randomization

A cluster randomized design will be used, since participants working together in the same unit are required to communicate and cooperate on certain assignments. The randomization procedure (1:1 ratio) will be carried out by a third party, i.e. the Unit for Applied Clinical Research at the Norwegian University of Science and Technology (https://www.ntnu.edu/mh/akf/randomisering). As the allocation of units to intervention or control arm will be concealed until all baseline measures have been completed, neither researchers nor participants will initially know to which arm they have been allocated. To ensure that a similar number of participants is assigned to the control and the intervention arm, the units will, during randomization, be stratified by their size (number of participants).

### Blinding

Due to the operations manager direct involvement in the intervention implementation after baseline, participant blinding will not be possible. Likewise, researcher blinding is unfeasible, as the researchers are involved in the delivery of the intervention.

## Methods – data collection, management and analysis

### Data collection methods

For an overview over assessment timeline see Table 1. Data will be collected at baseline before the intervention and again at the end of the intervention period. It is expected that the baseline measurements will be collected over a period of 6–8 weeks, but this phase may need to be extended if recruitment is slow. The intervention will thus commence once a sufficient number of participants is recruited and will continue until all post-intervention measurements have been completed. For the latter, 4 weeks are deemed sufficient, as all individuals will already be familiar with the procedures and no delays are anticipated. All collected data will be uploaded to a secure online storage system where it will be kept with access limited to the researchers conducting the study. All data will be pseudo-anonymized before analysis. In accordance with the regional ethical committee regulations, all data will be anonymized 5 years after the project is finalized, in 2028.

**Table 1:**
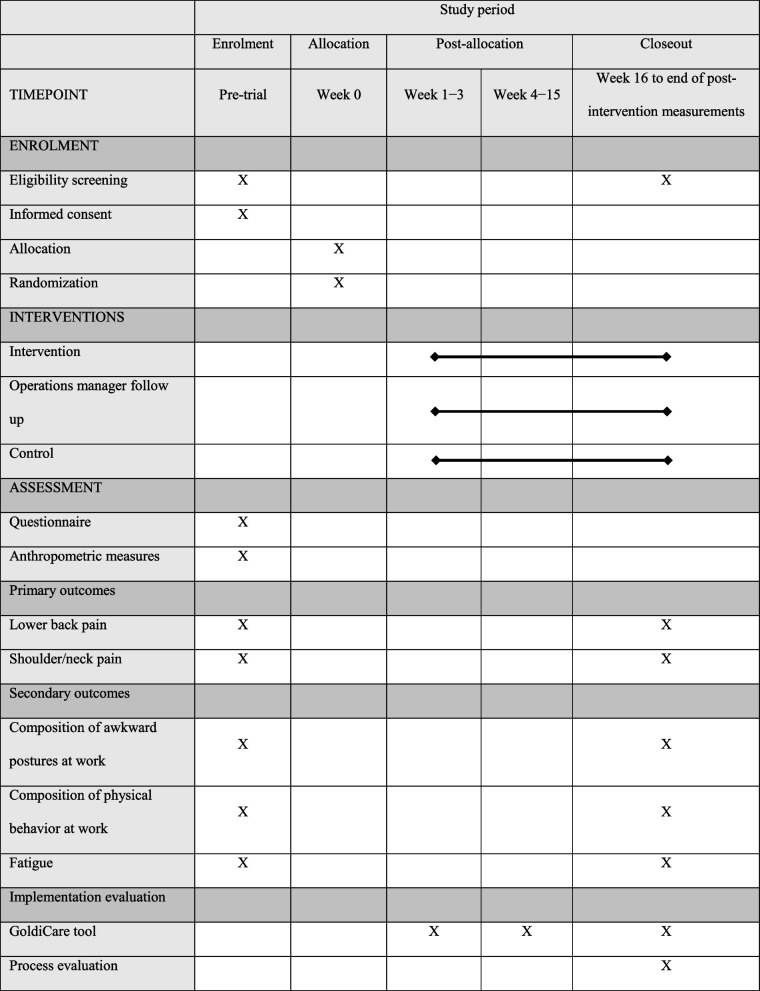
Schedule for enrolment, intervention and assessment in accordance with the SPIRIT statement [29]

#### Musculoskeletal pain

During both baseline and post-intervention measurements, participants will provide a lower back and neck/shoulder pain score when leaving the workplace. They will be instructed to select the numerical value on a 0 (no pain at all) to 10 (strong pain) scale [27] that most closely matches the level of pain experienced during the shift. They will be guided by the corresponding questions: “How much pain did you have in the lower back at the end of the working day?” and “How much pain did you have in the shoulder/neck at the end of this working day?”

#### Physical behavior

Each home care worker will be equipped with three Axivity AX3 (3-Axis Logging Accelerometer; Axivity Ltd., Newcastle upon Tyne, UK) accelerometers. One sensor will be placed on the thigh, approximately 10 cm above the patella, one will be placed on the upper back, at the T1 − T2 height, and the last one will be placed on the upper arm just below the deltoideus muscle. During measurements, participants will fill out an activity diary, indicating when they woke up, when they started their work shift, when it ended, and when they went to bed.

The initiation and setup of the AX3 accelerometers will be conducted in the Axivity software OmGui. The accelerometers will be set to record at ±8 g, at a frequency of 25 Hz for seven uninterrupted days. At the end of the recording period, the data will be downloaded from the accelerometers using OmGui and the acceleration signal will be processed in the MATLAB software Acti4 [30]. In Acti4, the acceleration signal is processed into physical behaviors including lying, sitting, standing, walking and running. In addition, Acti4 also detects the extent of trunk inclination and arm elevation. Thus, postures can be assessed for each of the physical behaviors [31]. Periods of work, leisure and sleep will be determined using the information in the activity diary.

While at work, home care workers will digitally register, in the home care registry, the start and end times of all assignments, which allows physical behavior to be analyzed specifically while the home care worker cares for patients with different ADL scores.

#### Anthropometric measures and questionnaire

During baseline measurements all participating home care workers will have their height (using a SECA 206 wall mounted measuring tape) and weight (using a standard bodyweight scale) measured, and will complete a questionnaire containing questions on demographics, socioeconomic status, self-rated health, and musculoskeletal pain. To assess the workplace psychosocial demands of the home care workers, pertinent scales from the Copenhagen Psychosocial Questionnaire III (COPSOQ III) will be adopted [32]. The scales will be “quantitative demands”, “work pace”, “emotional demands”, “influence”, “quality of leadership”, and “social support” from colleagues, as well as “social capital” (as a combination of vertical trust, longitudinal trust and organizational justice) [33].

#### GoldiCare tool

At the start of the intervention, each operations manager in the intervention group will receive an email with a link to a secure cloud server containing the GoldiCare tool. During the entire intervention period, operations managers will use the GoldiCare tool and store all entered data in the cloud server, allowing the researchers to check inputs, monitor compliance and provide regular feedback. To obtain a baseline for subsequent comparisons, the operations managers will be instructed to use the GoldiCare tool to record information for the week preceding the intervention.

#### Process evaluation

At the end of the intervention, operations managers will be invited to partake in qualitative semi-structured interviews to assess their compliance with the GoldiCare intervention and obtain their general perceptions of it. The home care workers will be invited to share their opinions of the intervention as a part of focus group discussions. While the goal of the focus groups is to ascertain whether home care workers found the intervention beneficial with respect to their health and productivity, the operations manager interviews will focus on the GoldiCare tool, how easy it was to use and its value in terms of efficiency in scheduling. All interviews and focus groups will be recorded. Two researchers will be present at the focus groups discussions.

### Statistical methods

To evaluate the primary outcomes, lower back pain and shoulder/neck pain in the intervention and the control group will be compared after the 16-week intervention, applying the intention-to-treat principle [34]. When conducting analyses, special considerations will have to be taken due to the clustered trial design [35, 36]. We will therefore adjust analyses for the effects of clustering by using mixed-model methods to account for differences between clusters while still providing insight into changes at the individual level [37]. Mixed-models will also be used to account for repeated measurements of pain intensity and fatigue at the individual level both at baseline and post-intervention. Parametric or non-parametric tests will be applied as appropriate.

Further, for physical behavior measures, compositional data analysis (CoDA) will be utilized as appropriate for time-use data [38, 39].

## Discussion

This protocol paper describes a cluster randomized controlled study evaluating the effectiveness of a Goldilocks Work intervention aiming to re-design allocation of tasks between home care workers in a home care unit, with the intention of improving musculoskeletal health. The home care sector will most likely have a greater influx of patients in the future, while at the same time facing problems in recruiting the necessary staff. Thus, better occupational health and a more health promoting work environment is urgently needed among home care workers, and we believe that the intervention described in the current protocol is a step in the right direction.

### Strengths and weaknesses

One of the main strengths of the intervention is its participatory approach, as involving home care workers and home care administration in the intervention development will ensure that it is feasible and appropriate for the current context. Moreover, an observational study was conducted to identify the focus for the intervention [2]. A feasibility study was also performed using a similar version of the GoldiCare tool to ensure that it is practical and easy to use for operations managers in home care. Further, this cluster randomized trial will be conducted in a real-life workplace setting, giving the intervention high ecological validity. As ADL scores are used nationwide, this intervention offers a possibility of changing the physical workload of home care workers on a national scale if proven effective. Finally, as participants are required to rate musculoskeletal pain after each workday throughout the week, the risk of recall bias is reduced, strengthening the value of the obtained findings.

However, it is important to note that the intervention has not been tested in a pilot investigation which would have been a strength. Also, we are aware that home care workers occasionally swap assignments, which will affect the validity of using the lists created by operations managers. Strict rules and a highly dynamic workplace make the home care sector especially challenging for conducting an intervention, but we believe this intervention is a push in the right direction.

## Data Availability

No data is produced in relation to the production of the present article, and sharing of data is therefore not applicable. Data produced following the protocol will not be publicly available, but may be made available upon reasonable request to the corresponding author. Results will be communicated in the form of scientific papers and in a report to the Norwegian Labour and Welfare Administration. Further, results will also be reported to relevant stakeholders (workers, management and cooperators from the municipality). Should important protocol changes occur, this will be made available in trial registries together with an explanation for any deviation from the protocol.

## Abbreviations

ADL: Activities of daily living
CoDA: Compositional data analysis
COPSOQ: Copenhagen Psychosocial questionnaire
